# Biodiversity of fungi in hot desert sands

**DOI:** 10.1002/mbo3.595

**Published:** 2018-03-05

**Authors:** Manuela Murgia, Maura Fiamma, Aleksandra Barac, Massimo Deligios, Vittorio Mazzarello, Bianca Paglietti, Pietro Cappuccinelli, Ahmed Al‐Qahtani, Andrea Squartini, Salvatore Rubino, Mohammed N. Al‐Ahdal

**Affiliations:** ^1^ Department of Biomedical Sciences University of Sassari Sassari Italy; ^2^ Clinic for Infectious and Tropical Diseases Clinical Centre of Serbia Faculty of Medicine University of Belgrade Belgrade Serbia; ^3^ Department of Infection and Immunity King Faisal Specialist Hospital and Research Centre Riyadh Saudi Arabia; ^4^ Department of Agronomy Animals, Food, Natural Resources and Environment DAFNAE University of Padova Padova Italy

**Keywords:** biodiversity, desert, fungi, sand, Saudi Arabia

## Abstract

The fungal community of six sand samples from Saudi Arabia and Jordan deserts was characterized by culture‐independent analysis via next generation sequencing of the 18S rRNA genes and by culture‐dependent methods followed by sequencing of internal transcribed spacer (ITS) region. By 18S sequencing were identified from 163 to 507 OTUs per sample, with a percentage of fungi ranging from 3.5% to 82.7%. The identified fungal Phyla were Ascomycota, Basal fungi, and Basidiomycota and the most abundant detected classes were Dothideomycetes, Pezizomycetes, and Sordariomycetes. A total of 11 colonies of filamentous fungi were isolated and cultured from six samples, and the ITS sequencing pointed toward five different species of the class Sordariomycetes, belonging to genera *Fusarium* (*F*. *redolens*,* F*. *solani*,* F*. *equiseti*), *Chaetomium* (*C. madrasense*), and *Albifimbria* (*A. terrestris*). The results of this study show an unexpectedly large fungal biodiversity in the Middle East desert sand and their possible role and implications on human health.

## 1 INTRODUCTION

Hot deserts are characterized by exceptionally limited availability of water and nutrients, extreme temperatures with wide day/night excursion, strong winds, and high ultraviolet (UV) radiation (Makhalanyane et al., 2015). Desert microbial communities are adapted to grow under hostile environmental conditions and have a foremost role in ecosystem bioprocesses (Pointing & Belnap, 2012). Compared to biomes of moderate or tropical regions, desert microbial communities differ in composition, function and display a low level of phylogenetic diversity (Fierer et al., 2012). Among eukaryotes, fungi are the most stress‐resistant organisms, able to grow in water‐scarcity and to tolerate desiccation through the formation of spores (Sterflinger, Tesei, & Zakharova, 2012). Fungi comprehend a heterogeneous group of saprobes, parasites or symbionts microorganisms and can be pathogens or endophytes (Gadd, 2007). Different groups of fungi are adapted to the desert environment, comprising terricolous fungi, fungi associated with plants, hyphomycetes, yeasts, and microcolonial fungi (Sterflinger et al., 2012).

Although molecular studies of fungal diversity have started to emerge in recent years (Buée et al., 2009; Jumpponen, Jones, David Mattox, & Yaege, 2010; Zimmerman & Vitousek, 2012), the fungal communities of deserts are still poorly uncovered, especially with respect to human pathogenic fungi. In fact, desert mycological studies are mainly based on culture‐dependent methods, which detect only a fraction of the fungal community (Sterflinger et al., 2012). As deserts are frequently visited by tourists and locals, the presence of pathogenic fungi might pose a risk to human health. Moreover, fungi that are usually saprophytes may act as opportunistic pathogens, especially in immunocompromised patients, or as allergens in atopic hosts (De Hoog et al., 2000). Furthermore, fungi and bacteria can be transported for long distance by the desert dust movement, including trans‐pelagic migrations across continents, posing concerns for human health (Goyer, 2004; Rosselli et al., 2015; Sano et al., 1997).

The geography of Saudi Arabia and Jordan is mostly represented by the Arabian deserts, associated semi‐deserts and scrubland. The climate of Saudi Arabia deserts is represented by extremely high daytime temperatures and an extreme temperature drop during nights, while Jordan has moderate temperature. Average summer temperature in Saudi Arabia is around 45°C but maximum values could reach 54°C, while the average temperature in Jordan is 20–35°C. The amount of annual rainfall is very low in these countries (Peel, Finlayson, & McMahon, 2007). Internationally, these sites are recognized for their natural and geomorphological value and have high conservation value for research and eco‐tourism.

So far, only dated culture‐based studies have been carried out on Saudi Arabia desert fungi (Abdel‐hafez, 1981; Abdel‐Hafez, 1982a,b,c,d), whereas the fungal communities of Jordan deserts have not yet been investigated. Therefore, the aim of this study was to improve the knowledge of the biodiversity of the filamentous fungi in these deserts sand by culture‐dependent and independent methods. The identification of these fungi could be useful in the recognition of new sources for bioactive metabolites and in the determination of hazards posed by desert fungi on the development of human infection diseases.

## 2 MATERIALS AND METHODS

### 2.1 Sampling sites

**Figure 1:**
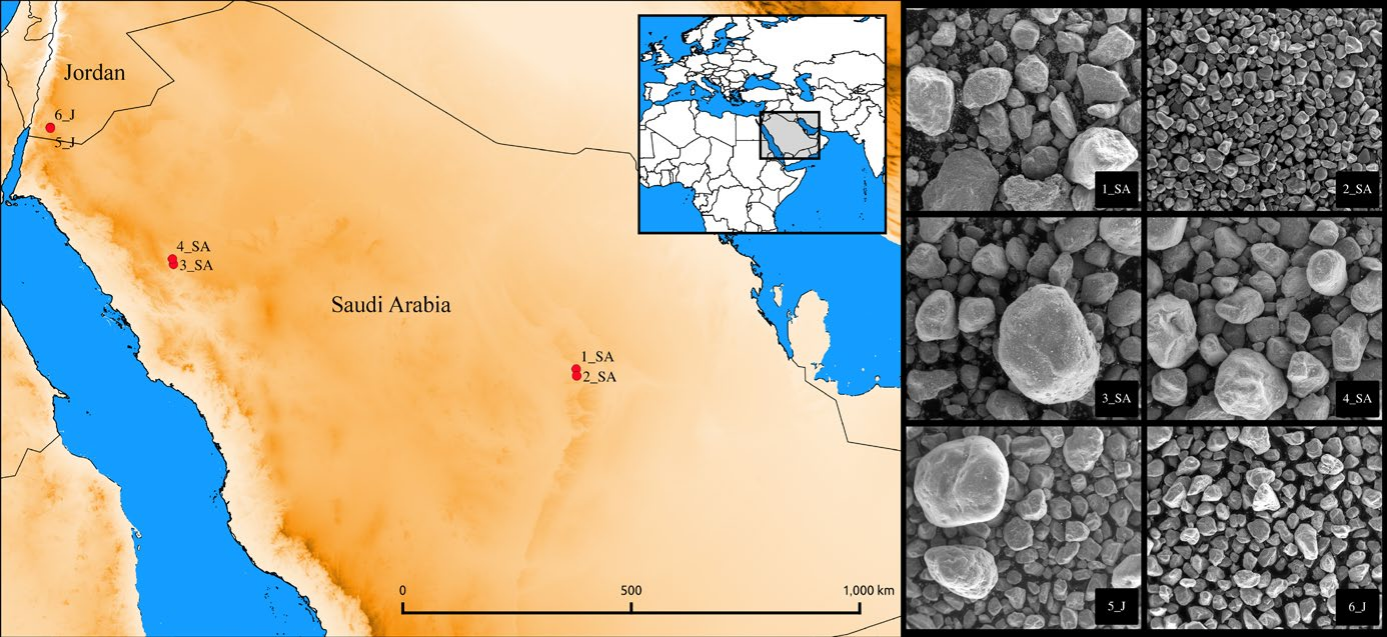
Left: Sites of sampling. Map was generated with QGIS 2.8.6‐Wien (qgis.org) using vector and raster data from http://www.diva-gis.org/Data. Right: SEM images of the sands. SA, Saudi Arabia; J, Jordan

Overall six sand samples were collected, four from Saudi Arabia and two from Jordan deserts. In detail, two samples were collected in Central Saudi Arabia (Riyadh Province) from the White desert in Al‐Melaiheyyah (1_SA) and from the Red desert in Rawdat Al‐Mahalleyah (2_SA) in. Two samples were collected in Northern Saudi Arabia from the Elephant rock site in Al Ula (3_SA) and from the archeological site of Mada'in Saleh (4_SA). The last two samples were collected in Southern Jordan from two different locations in Wadi Rum desert (5_J and 6_J) (Figure 1, Table 1). Sampling sites were open deserts without plants, and only sample 1_SA was collected in a zone with plants, close to an urban settlement. Sampling sites of 3_SA and 4_SA were important tourist areas. Surface sand samples were taken using sterile labeled 50 ml Falcon tubes and sterile gloves, placed in plastic bags and transported to the Department of Biomedical Sciences of the University of Sassari, Sardinia, Italy.

**Table 1:** Sampling sites, geographical coordinates, date of sampling, and monthly mean temperature (*T*_mean_)

Sample name	Place of sampling		Coordinates	Date	*T*_mean_
	Country	Site			
1_SA	Saudi Arabia	White desert (Al‐Melaiheyyah, Riyadh)	24.51°N 46.35°E	June 2013	27–41°C
2_SA		Red desert (Rawdat Al‐Mahalleyah, Riyadh)	24.37°N 46.37°E	January 2014	8–17°C
3_SA		Elephant rock site (Al Ula, Mada'in Saleh)	26.69°N 37.98°E	May 2013	20–43°C
4_SA		Archaeological site (Mada'in Saleh)	26.8°N 37.96°E	May 2013	20–43°C
5_J	Jordan	Wadi Rum desert	29.52°N 35.42°E	January 2014	9–20°C
6_J		Wadi Rum desert	29.54°N 35.42°E	January 2014	9–20°C

SA, Saudi Arabia; J, Jordan.

### 2.2 Scanning electron microspopy

Fine grained sediment was mounted on aluminum stubs and viewing a low vacuum in a FEI QUANTA 200 SEM at 20 keV beam accelerating voltage, 0° tilt angle and 17 mm working distance. The image analysis program (ImageJ 1.38e) was used to measure the grain size of sand (Prakongkep, Suddhiprakarn, Kheoruenromne, & Gilkes, 2010).

### 2.3 Culture‐independent 18S sequencing analysis

One gram of each sand sample was aseptically weighed and subjected to total DNA extraction using the E.Z.N.A.^®^ Soil DNA Kit according to the manufacturer's protocol (Omega Bio‐tek Inc, Norcross, GA, USA). DNA concentration and quality were measured with a NanoDrop 2000 spectrophotometer (Thermo Fisher Scientific Inc.). 18S rRNA was amplified using the universal eukaryote primers 18S‐82F (GAAACTGCGAATGGCTC) and Ek‐516r (ACCAGACTTGCCCTCC) (Casamayor et al., 2002; López‐García, Philippe, Gail, & Moreira, 2003). PCR reactions were carried‐out using Platinum^®^ Taq DNA Polymerase High Fidelity (Life Technologies) on a PTC‐200 Thermal Cycler (MJ Research Inc.) with the following conditions: initial denaturation at 95°C for 5 min, followed by 30 cycles of 95°C for 30 sec, 51°C for 30 sec, 72°C for 2 min, and final extension at 72°C for 10 min. Amplicons were purified with DNA Clean & Concentrator kit (Zymo Research) and an aliquot of DNA (5 μl at 0.2 ng/μl) was sequenced at the Porto Conte Ricerche S.r.l. (Alghero, Italy) using the Illumina HiScanSQ platform. Paired‐end sequences were merged and filtered using Usearch (Edgar, 2010) with the following parameters: fastq_truncqual 15, fastq_minovlen 8, fastq_maxdiffs 0 and fastq_minlen 100. Operational Taxonomic Unit (OTU) picking at 97% of similarity was performed using QIIME 1.8.0 using the Silva release 111 database (Caporaso et al., 2010).

#### 2.3.1 Venn diagrams and biodiversity indices

Sequences were categorized by aspects of interest, analyzed, and displayed by use of Venny 2.161 (http://bioinfogp.cnb.csic.es/tools/venny/index.html). The alpha and beta diversity was calculated using QIIME. In particular, were calculated Shannon and Chao1 indexes plus the observed OTUs for alfa diversity. The samples were also clustered in a tree using Unweighted Pair Group Method with Arithmetic mean (UPGMA) for beta diversity.

### 2.4 Isolation and identification of filamentous fungi

An amount of 0.1 g of each sand sample was aseptically weighed and inoculated into plates of Sabouraud dextrose agar (SDA) with cloramphenicol (0.05 g/L) and gentamicin (0.1 g/L) (Microbiol. diagnostici, Uta, Italy). Plates were incubated at room temperature for 7–15 days. Identification of fungi was done based on the macroscopic and microscopic characteristics using standard mycological methods (Hawksworth et al., 1995). DNA of isolated fungi was extracted from homogenized samples (mycelium) using MagPurix Plant DNA extraction kit (Zinexts Life Science Corp, New Taipei City, Taiwan) with the automatic extractor SaMag^™^ System (Sacace Biotechnologies, Como, Italy), as described by the manufacturer. Molecular species identification was performed by PCR with fungal‐specific primers ITS1F and ITS4 (White, Bruns, Lee, & Taylor, 1990) targeting the internal transcribed spacer (ITS) region of the ribosomal rRNA gene with the following conditions: initial denaturation at 94°C for 5 min, followed by 30 cycles of 94°C for 1 min, 50°C for 90 s, 72°C for 90 s, and final extension at 72°C for 7 min. PCR products were purified using QIAquick PCR purification Kit (Qiagen, Hilden, Germany) and sequenced (CERSAA, Albenga, Italy). The sequences were aligned with BLAST against the GenBank database (Altschul, Gish, Miller, Myers, & Lipman, 1990). Sequences were deposited in NCBI (acc. numb: MG679518‐24). Moreover, the ITS sequences obtained were clustered with reference sequences and it was built a neighbor joining phylogenetic tree using Geneious 4.8.5 with 1000 bootstrap replication ((http://www.geneious.com, Kearse et al., 2012)

## 3 RESULTS

### 3.1 Scanning Electron Microscopy

Scanning Electron Microscopy (SEM) observation of the six desert sand samples showed that the sands can be classified according to ISO 14688‐1:2002 as “Coarse soil” with fine, medium, and coarse sand depending on the site (Figure 1). Samples 2_SA from Red desert (Riyadh Province) and 6_J from Wadi Rum were constituted of fine, regular sand grains with size average of 234 ± 89 μm and 384 ± 105 μm, respectively. Samples 4_SA from Mada'in Saleh and 5_J from Wadi Rum were composed of medium sand with irregular appearance and grain size average of 660 ± 201 μm and 773 ± 372 μm, respectively. Samples 1_SA from White desert (Riyadh Province) and 3_SA from Al Ula consisted of coarse, irregular sand grains with size average of 1.110 ± 420 μm and 1.410 ± 398 μm, respectively (Figure 1).

### 3.2 Richness and diversity of fungi by culture‐independent 18S sequencing analysis

The presence of fungi was detected by 18S sequencing analysis in all the six samples analyzed.

**Figure 2:**
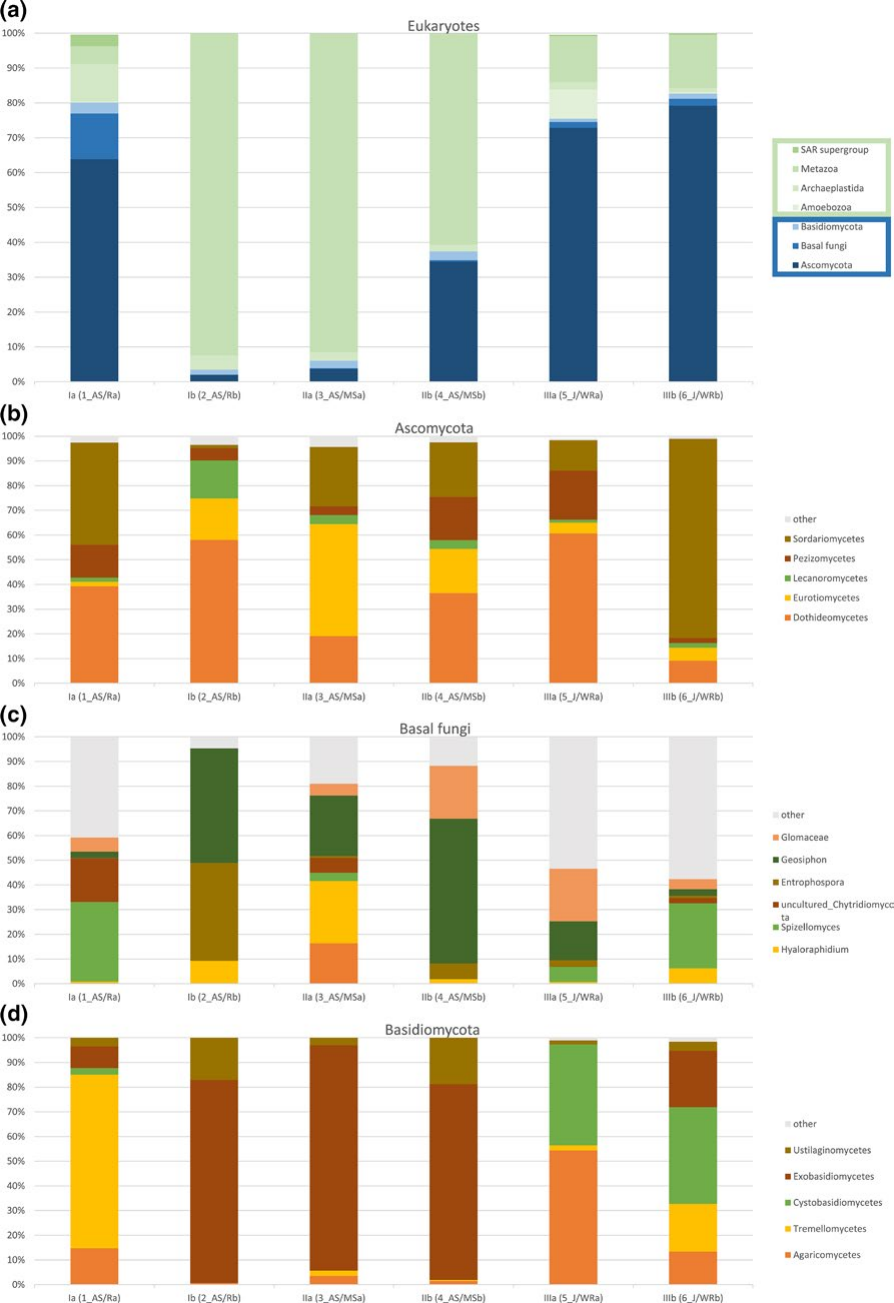
Percentage of aligned sequences in each sand sample. (a) All eukaryotic phyla; (b) classes of the phylum Ascomycota; (c) classes of the phylum Basal Fungi; (d) classes of the phylum Basidiomycota. SA, Saudi Arabia; J, Jordan

Fungi represented from 75.4% to 82.7% of the eukaryotes identified in Jordanian samples. Instead, in the samples collected from Saudi Arabia deserts the presence of fungi ranged from 3.5% to 80%. Other eukaryotes detected were mainly metazoa, archeoplastidia, and amebozoa (Figure 2a). The most common fungal Phyla identified in all the samples were Ascomycota, followed by Basal fungi and Basidiomycota (Figure 2). A complete list of the OTUs can be browsed as spreadsheet in Table S1 online.

#### 3.2.1 Saudi Arabia deserts

In sample 1_SA, collected from the White Desert of Riyadh, 80% of eukaryotic DNA was represented by fungi, whereas 11% belonged to Archeaplastidia, 5% to Metazoa and 3% to SAR supergroup. Among fungi, 64% belonged to the Phylum Ascomycota, 13% to Basal fungi and 3% to Basidiomycota. The predominant fungal classes identified among Ascomycota were Sordariomycetes (26.42%), Dothideomycetes (25.08%) and Pezizomycetes (8.5%), followed by Spizellomyces (4.25%) and Chytridiomycota (2.33%) among Basal fungi and by Tremellomycetes (2.2%) among Basidiomycota (Figure 2). In this sample DNA of Basal fungi from the genus *Triparticalcar* (0.24%) was detected, which was absent in other samples.

Eukaryotes detected in sample 2_SA from Red desert of Riyadh were mainly Metazoa (92.4%) and Archaeplastidia (4.1%). Fungi were only 3.5%, 2% of which Ascomycota, 1.5% Basidiomycota, and 0.03% Basal fungi. The classes mainly represented were Exobasidiomycetes (1.2%) and Dothideomycetes (1.2%).

In sample 3_SA, collected from Elephant rock in Al Ula, eukaryotes were mainly represented by Metazoa (91.4%), whereas fungi were 6%, of which 3.7% Ascomycota, 2.1% Basidiomycota, and 0.2% Basal fungi. The most represented class among Ascomycota was Exobasidiomycetes (2%). Eukaryotes detected in sample 4_SA from the archeological site of Mada'in Saleh were mainly Metazoa (60.5%) followed by fungi (37.4%). The most common fungal Phylum was Ascomycota (34.5%), while Basal fungi and Basidiomycota were less represented (0.3% and 2.5%, respectively). In this sample the classes Dothideomycetes (12.6%), Sordariomycetes (7.6%), Eurotiomycetes (6.2%), Pezizomycetes (6%), belonging to the subphylum Pezizomycotina were detected (Figure 2).

#### 3.2.2 Jordan desert

In sample 5_J from Wadi Rum desert, 75.4% of the eukaryotic DNA was represented by fungi. Other Eukaryotes detected were Metazoa (13%), Amebozoa (8.5%), and Archeaplastidia (2%). The most common fungal Phylum was Ascomycota (72.8%), while the presence of DNA of Basal fungi and Basidiomycota was low (1.7% and 1%, respectively). The subphylum Pezizomycotina was the most represented and included the classes Dothideomycetes (44.2%), Pezizomycetes (14.5%), Sordariomycetes (9%), and Eurotiomycetes (3%). Sample 6_J displayed the highest presence of fungal DNA (82.7%), whereas other Eukaryotes were mainly Metazoa (15.2%). The most common fungal Phylum was Ascomycota (79.2%), followed by Basal fungi (2%) and Basidiomycota (1.5%). The most represented classes were Sordariomycetes (64%), Dothideomycetes (7.2%), Eurotiomycetes (4.2%), Pezizomycetes (1.6%), and Lecanoromycetes (1.5%) (Figure 2).

#### 3.2.3 Eukaryotic richness and diversity in the sand samples

**Figure 3:**
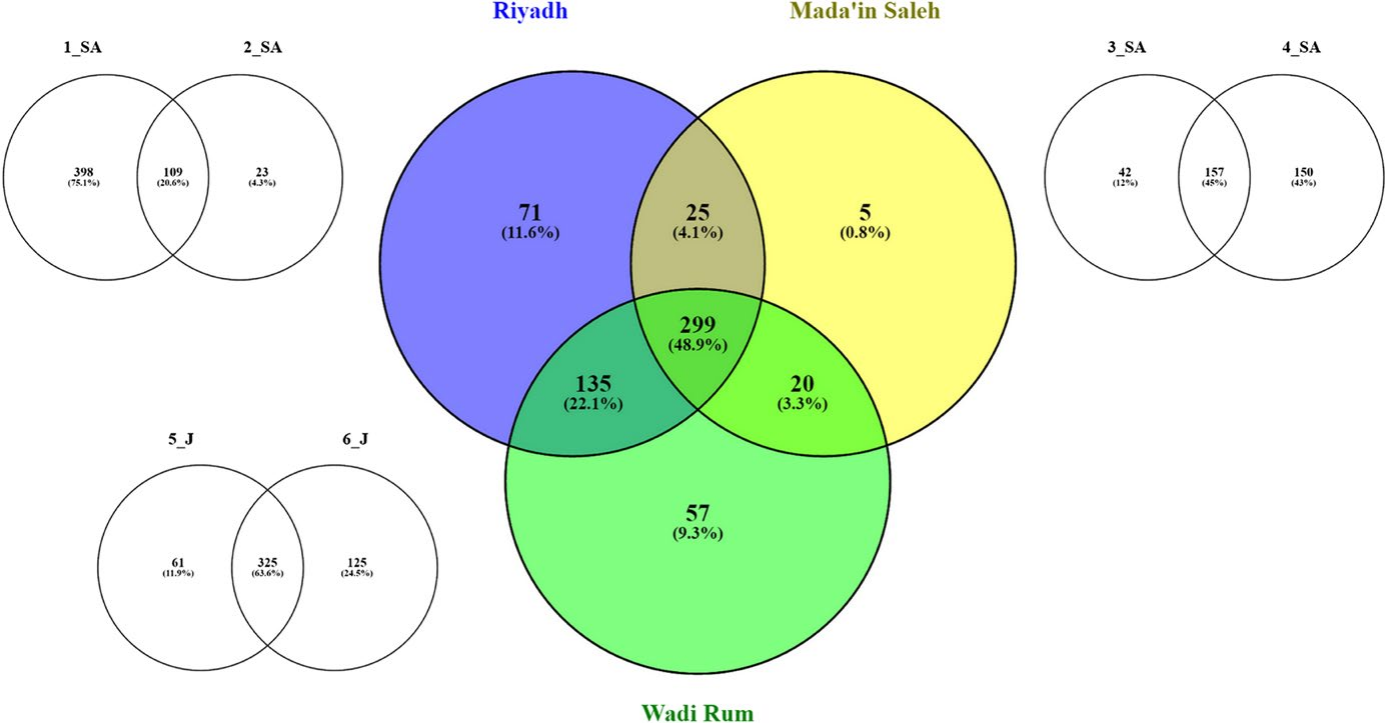
Venn diagram showing the number of specific and shared genera identified in each of the six samples. For each location, a diagram of overlaps between the two samples is shown in colors. SA, Saudi Arabia; J, Jordan

**Figure 4:**
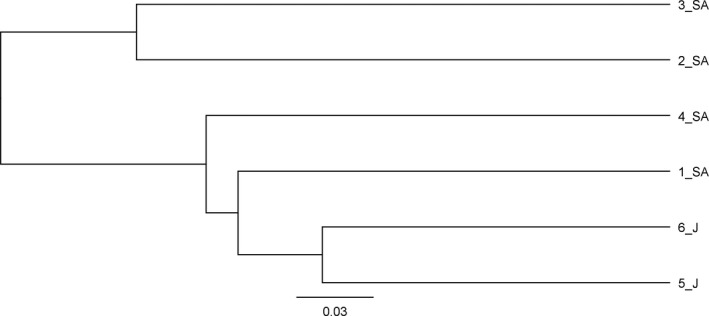
UPGMA tree inferred from 18S rRNA genes of the six desert sand samples

A total of 831 OTUs were identified in all the six samples, ranging from 163 in sample 2_SA to 507 in samples 1_SA and 6_J (Table 2). We compared the richness calculated by the Chao1 estimator, which ranged from 212 to 636 OTUs per sample. Moreover, the measured diversity, estimated using the Shannon index, ranged from 4.0 to 6.8 per sample. All these alpha diversity indices showed that the sample with the highest diversity level was 1_SA, while the sample with the lowest diversity level was 2_SA, both collected in the Riyadh Province (Table 2). Venn diagrams were calculated on shared OTUs between the three locations: Riyadh (samples 1_SA and 2_SA), Mada'in Saleh (3_SA and 4_SA), and Wadi Rum (5_J and 6_J). The prevailing occurrence of a common core of shared taxa is shown in detail by the overlapping Venn diagrams representation in Figure 3. The highest proportions of taxa were those shared by all three locations: 299 (48.9%), followed by 71 (11.6%) from Riyadh, and by 135 (22.1%) found in both the locations of Riyadh and Wadi Rum, while the least shared level of taxa was found in Mada'in Saleh with a value of 5 (0.8%). Comparing the samples, the high percentage of specific taxa was found in sample 1_SA from Riyadh with 398 (75.1%), followed by sample 4_SA from Mada'in Saleh with 150 (43%). Regarding the beta diversity, UPGMA tree showed that Jordan samples clustered together, whereas Arabian samples were not grouped according to the sampling site (Figure 4).

**Table 2:** Alpha diversity, species, OTU richness, and number of sequences of each desert sand sample

Sample	1_SA	2_SA	3_SA	4_SA	5_J	6_J
Shannon^a^	6.8	4.0	4.1	5.6	5.4	6.1
Chao1^a^	636.6	212.8	311.6	449.8	534.1	606.0
Species^a^	613.9	184.4	272.8	399.1	481.7	564.7
OTUs^a^	507	163	229	355	423	507
Number of sequences	317109	125050	142652	277801	272781	405147

SA,Saudi Arabia; J,Jordan.

a Normalized at 88,197 reads.

### 3.3 Isolation and taxonomic characterization of fungi

**Figure 5:**
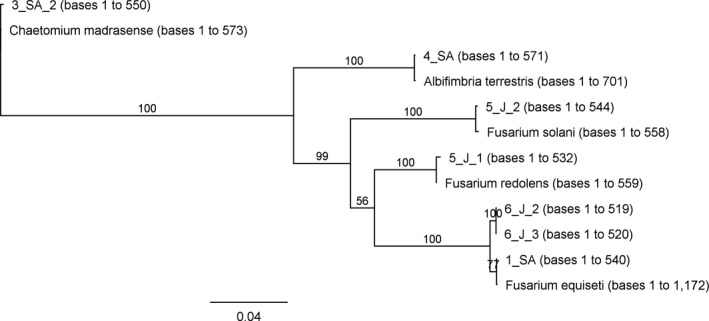
Phylogenetic tree of ITS sequences of our isolates and reference sequences

On the basis of cultivable approach, 11 filamentous fungi were isolated from the six desert sand samples. Three colonies grown from both the Jordanian samples, two colonies grown from the Saudi Arabian sample 3_SA, and three colonies grown from three other remaining Arabian samples. ITS sequencing allowed to trace the presumed identities (expressed as top‐scoring homologies to GenBank records) of seven filamentous fungi, all belonging to the class Sordariomycetes. *Fusarium* was the most represented genus with three bona fide species, followed by *Chaetomium* (one species) and *Albifimbria* (one species), as showed in Table 3. Four isolates (not listed in Table 3) were not identified. A phylogenetic tree constructed using the ITS sequences obtained showed that our isolates clustered with the correspondent reference species (Figure 5).

**Table 3:** Fungal species identified by culture in desert samples

Isolate	Genbank code	Best‐match homologies to GenBank subjects	Identity level (%)	Query coverage (%)	E‐Value
1 SA	MG679518	*Fusarium equiseti* (KR094457)	100	100	0.0
3 SA 2	MG679519	*Chaetomium madrasense* (KT192312)	100	100	0.0
4 SA	MG679520	*Albifimbria terrestris* (KU845884)	99.82	100	0.0
5 J 1	MG679521	*Fusarium redolens* (HQ703404)	100	100	0.0
5 J 2	MG679522	*Fusarium solani* (KX343148)	100	100	0.0
6 J 2	MG679523	*Fusarium equiseti* (KY365589)	99.59	100	0.0
6 J 3	MG679524	*Fusarium equiseti* (KY365589)	99.60	100	0.0

SA, Saudi Arabia; J, Jordan.

## 4 DISCUSSION

The life‐limiting hot desert sands of the Middle East represent a harsh but extremely extended facet of our planet. In this study, we sampled a transect of three locations from Jordan to Central Saudi Arabia (Wadi Rum, Mada'in Saleh and Riyadh), exploiting for the first time eukaryotic population diversity of surface desert sand samples, with particular reference to fungal communities, using a metagenomic DNA sequencing approach as well as culture‐based analyses.

A total of 612 taxa were detected with 18S rRNA sequencing analysis. In three of the samples analyzed, fungi represented more of the 75% of the eukaryotes identified, suggesting their possible ecosystemic role within the microbial biodiversity of the desert environment. The most abundant fungal Phylum identified in all the samples analyzed was Ascomycota and the prevalent classes were Dothideomycetes, Sordariomycetes, and Pezizomycetes. Dothideomycetes is the largest and most ecologically diverse class of fungi with important roles in ecosystem and carbon cycling as degraders of plant biomass (Kirk, Cannon, Minter, & Stalpers, 2008). Dothideomycetes are present on every continent, even Antarctica (Ruibal et al., 2009; Selbmann, Grube, Onofri, Isola, & Zucconi, 2013); many are tolerant to environmental extremes including very high or low temperatures, dehydration and solar radiation. Many species are plant pathogens or saprobes and only few species are pathogens of humans or animals (Gioulekas et al., 2004). The prevalence of Dothideomycetes in our samples could be related with their capability to survive in extreme conditions and suggests that this class of fungi plays a significant role in the desert ecosystem. Among Dothideomycetes we detected the Genus *Alternaria,* of which many species are saprophytes and most are known due to their impact as animal and plant pathogens (De Hoog & Horré, 2002). *Alternaria* is one of the most common air allergens in immunocompetent patients, while it is among the most common causative agents of phaeohyphomycosis in immunocompromised patients (De Hoog & Horré, 2002; Gioulekas et al., 2004; Ohm et al., 2012).

The class Sordariomycetes includes a number of important plant pathogenic species, saprophytes, as well as important human pathogens (Hoog et al., 2000). The genus *Fusarium* was also detected in our sand samples. It is reported that some species as *Fusarium* may cause nail and cornea infections in immunocompetent individuals, while disseminated infections are seen in immunocompromised patients (Dignani & Anaissie, 2004).

Pezizomycetes is a diverse class with a role in ecological processes and symbioses including animal and plant pathogens and existing in aquatic and arid environments, but mostly occurring in hot regions or at high altitudes (Spatafora et al., 2006). As confirmed in our study, this class of fungi is common in tropical regions (Hansen & Pfister, 2006).

The metagenomic analysis also detected some interesting fungi belonging to the class Zoopagomycotina, order Cochlonema, free‐living amoebas and zooparasites, and fungi belonging to the class Mucormycotina. Although their DNA was not isolated in high percentage in sand samples, any isolate of this class deserves discussion. Detection of DNA belonging to the basal fungi, class Mucormycotina, order Mortierellales is also of great importance. To the best of our knowledge, only two reports of human infection with *M. wolfii* have been published yet, especially as *Mortierella* is known as a dominant zoo pathogen (Layios, Canivet, Baron, Moutschen, & Hayette, 2014). There are new studies about the role of *Mortierella* on the development of human diseases. The order Mucorales was also detected in our sand samples. It comprises 240 described species, while at least 20 have been found to be involved in mucormycosis. Especially the order Mucorales of the Mucoromycotina encompasses several human pathogenic species. Although infections with mucoralean fungi (mucormycosis) are less common than aspergilloses, these fungi are increasingly recognized as the source of fulminant infections in immunocompromised patients. Mucormycoses are associated with rapid blood vessel invasion, necrosis, and massive destruction of tissue with high mortality rate (70–100%) (Chakrabarti et al., 2006; Skiada et al., 2011).

In this study, as the Venn diagrams analysis allows to appreciate, it appears that almost half of the OTU diversity (49%) was shared among the three locations (Riyadh, Mada'in Saleh and Wadi Rum) and thus represents a common ubiquitous core of widespread species occurring across the whole Arabian peninsula and Southern Jordan. As regards the other non‐shared taxa, it is interesting to notice that distance is not playing a hierarchically‐dominant role as there is a wider overlap (22.1%) between the two distant sites in Riyadh and Wadi Rum than the one observed between each of these extremes with the Mada'in Saleh location which lies in the middle, about halfway from each, and with which they share only 4.1% and 3.3 %, respectively. In the same way, the three locations were no correlated with the sand granulometry. For instance, the grain size of the sands form the Riyadh province (samples 1_SA and 2_SA) was different, coarse, and fine, respectively.

The UPGMA tree showed that samples from Wadi Rum desert in Jordan were closer in respect with the others. Moreover, both of them displayed high amount of fungal DNA. This can be due to the moderate temperatures and humidity at the time of sampling that creates the ideal conditions for fungal growth. Instead, Arabian samples showed less quantity of fungi, apart for sample 1_SA from White Desert near Riyadh. The reason could be that this sample was collected in a zone with plants, frequented by animals and humans. Riyadh is an extensive industrial zone with many crossroads where air may contain particles from different sources which occasionally could converge due to sandstorms (Tazaki et al., 2004). By that way, opaque masses of air dust containing sand particles and fungi could be transported by strong winds and assume a role in the spread of fungal diseases (Rosselli et al., 2015). As the sand from deserts surrounding populated places could be a source for mycoses, the establishment of fungal biodiversity in sand is of profound epidemiological importance.

It has been suggested that the presence of fungi is related to direct or indirect contamination originating from organic sources and climate variations. The principal microbial risk to human health encountered with sand in desert is similar and arising from contact with animal excreta, mostly camels, used as touristic attraction, or other smaller desert animals.

As expected, we gained more information about fungal communities in desert sand by metagenomic analysis compared to traditional culturable method. However, with the culturable method we were able to isolated several fungi whose ecology is linked to plants. *Fusarium* was the most abundant fungal genus isolated and three different species were identified. It needs to be remarked that the taxonomy of the *Fusarium* genus presents issues of difficult resolution when based on the ITS sequence. For this reason, our attributions for this genus should be considered as the best matches resulting from BLAST alignments against the NCBI database. However, besides the criterion of 100% identity and 100% query coverage, the attributed taxa have been selected on the basis of their most consistently recurring nominal consensus among all the equally matching taxa. *F. redolens* and *F. equiseti* were signaled as causal agents of Aleppo pine damping off in Algeria (Lazreg et al., 2013). The *F. solani* complex, besides being a common pathogen causing wilt in different accounts for about 50% of human infections caused by *Fusarium* (Guarro, 2013; Salah et al., 2015; Tortorano et al., 2014). The genus *Chaetomium*, together with more common genera *Penicillium* and *Trichoderma,* belong to phylum Ascomycota (Wang, Houbraken, groenewald, Meijer, et al., 2016). *Chaetomium* is cosmopolitan species occurs in soil, dung, wide plant materials, air and marine environments (Wang, Lombard, Groenewald, Li, et al., 2016). *Chaetomium* has also been reported to directly infect humans, and is most commonly associated with onychomycosis, but there is also one report from Indian authors presenting the case of corneal ulcer due to a coelomycetes (Kim et al., 2013; Reddy, Venugopal, Prakash, & Kamath, 2017). Members of family *Stachybotriaceae* include important plant and human pathogens, as well as several species used in industrial and commercial applications as biodegrades and biocontrol agents. Previous study of multi‐locus phylogenetic showed that the genera *Myrothecium* is polyphyletic resulting in the introduction of 13 new genera with *Myrothecium*‐like morphology. Among others, previously known all under the name of *Myrothecium* species, *Albifimbria* is for the first time recognized as independent species with distinct morphology (Lombard et al., 2016). However, the impact of *Albifimbria* presence in sand and its implication on the human health is still insufficiently researched.

In conclusion, our results evidence an unexpectedly large fungal biodiversity in the Middle East desert sand, predominantly consisting of fungi adaptive for survival in the extreme environmental conditions prevailing in deserts, indicating the robustness and endurance of microorganisms such as the fungal classes Dothideomycetes, Pezizomycetes, and Sordariomycetes, which are worthy of further investigation. This study suggests that desert sand and plants may be a rich source of fungi, tolerant to extreme environmental conditions that could be examined for plant growth promotion activity in agriculture. Moreover, this study might be helpful to resolve the strategies adopted by microbes in response to extreme conditions to defeat draining by induction of various types of solutes. Furthermore physico‐chemical analyses of sand could uncover a more complete screenplay of the abiotic factors and fungal interplay conditions existing in the desert areas.

## CONFLICTS OF INTEREST

Authors declare that they have no conflicts of interest.
